# Multispectral Imaging for Metallic Biopsy Marker Detection During MRI-Guided Breast Biopsy: A Feasibility Study for Clinical Translation

**DOI:** 10.3389/fonc.2021.605014

**Published:** 2021-03-22

**Authors:** Sarah Eskreis-Winkler, Katherine Simon, Melissa Reichman, Pascal Spincemaille, Thanh D. Nguyen, Paul J. Christos, Michele Drotman, Martin R. Prince, Katja Pinker, Elizabeth J. Sutton, Elizabeth A. Morris, Yi Wang

**Affiliations:** ^1^ Department of Radiology, Weill Cornell Medicine, New York, NY, United States; ^2^ Department of Radiology, Memorial Sloan Kettering Cancer Center, New York, NY, United States; ^3^ Division of Biostatistics and Epidemiology, Department of Healthcare Policy & Research, Weill Cornell Medicine, New York, NY, United States

**Keywords:** magnetic resonance imaging (MRI)-guided breast biopsy, biopsy marker, multispectral imaging, breast magnetic resonance imaging (MRI), mammography

## Abstract

**Purpose:**

To assess the feasibility and diagnostic accuracy of multispectral MRI (MSI) in the detection and localization of biopsy markers during MRI-guided breast biopsy.

**Methods:**

This prospective study included 20 patients undergoing MR-guided breast biopsy. In 10 patients (Group 1), MSI was acquired following tissue sampling and biopsy marker deployment. In the other 10 patients (Group 2), MSI was acquired following tissue sampling but before biopsy marker deployment (to simulate deployment failure). All patients received post-procedure mammograms. Group 1 and Group 2 designations, in combination with the post-procedure mammogram, served as the reference standard. The diagnostic performance of MSI for biopsy marker identification was independently evaluated by two readers using two-spectral-bin MR and one-spectral-bin MR. The κ statistic was used to assess inter-rater agreement for biopsy marker identification.

**Results:**

The sensitivity, specificity, and accuracy of biopsy marker detection for readers 1 and 2 using 2-bin MSI were 90.0% (9/10) and 90.0% (9/10), 100.0% (10/10) and 100.0% (10/10), 95.0% (19/20) and 95.0% (19/20); and using 1-bin MSI were 70.0% (7/10) and 80.0% (8/10), 100.0% (8/8) and 100.0% (10/10), 85.0% (17/20) and 90.0% (18/20). Positive predictive value was 100% for both readers for all numbers of bins. Inter-rater agreement was excellent: κ was 1.0 for 2-bin MSI and 0.81 for 1-bin MSI.

**Conclusion:**

MSI is a feasible, diagnostically accurate technique for identifying metallic biopsy markers during MRI-guided breast biopsy and may eliminate the need for a post-procedure mammogram.

## Introduction

Breast magnetic resonance imaging (MRI) is used increasingly for both cancer screening in high-risk women and extent-of-disease evaluation in newly-diagnosed breast cancer patients (1). Breast MR often detects lesions which are not visible on mammogram and ultrasound, which in turn has increased the demand for MRI-guided breast biopsy (2). During biopsy, suspicious lesions are identified with dynamic contrast-enhanced (DCE)-MRI, tissue samples are obtained, and a small metallic biopsy marker is deployed into the biopsy cavity to enable lesion localization on conventional imaging, such as mammography and sonography, and to guide future interventions (3). However, conventional MRI is unable to distinguish titanium biopsy markers from the surrounding air that is often introduced into the breast during biopsy. Both titanium and air have high magnetic susceptibility and appear as signal voids due to dephasing (4). In order to establish that a metallic biopsy marker has been successful deployed and is properly positioned, a post-procedure mammogram is routinely obtained.

Using a short MR protocol instead of a mammogram for biopsy marker detection and localization would yield several advantages. First, it would permit biopsy marker evaluation during the biopsy procedure, allowing for real-time deployment of a second biopsy marker if the first one fails to deploy or is not identified otherwise (5, 6). Second, it would obviate the need for a post-procedure mammogram, which is inconvenient, uncomfortable (especially after breast biopsy), and involves ionizing radiation. Additionally, it would replace the 2D mammographic projection image with 3D cross-sectional information to more precisely pinpoint the biopsy marker location.

Multispectral imaging (MSI) techniques (e.g., SEMAC and MAVRIC) are commonly used for metal artifact reduction (7–12) but can be modified to distinguish biopsy markers from surrounding air by selectively exciting the metallic marker’s magnetic field isocontours. MSI is generally performed with a large number of spectral bins (i.e., 24 bins) resulting in a relatively long scan time. However, since the magnetic field isocontours generated by biopsy markers are known, we employed a reduced-bin MSI (i.e., 2 bins) in an effort to shorten the scan time of this technique and to allow easy integration into a routine MRI breast biopsy protocol.

The purpose of this pilot study is to investigate the feasibility and diagnostic performance of reduced bin MSI for the detection and localization of biopsy markers after MRI-guided breast biopsy.

## Materials and Methods

This prospective study was approved by our Institutional Review Board and was compliant with the Health Insurance Portability and Accountability Act. Written informed consent was obtained from all patients before MRI-guided breast biopsy.

### Patient Population

Twenty-two female patients undergoing MRI-guided breast biopsy for a suspicious finding on diagnostic MRI (BI-RADS 4 or 5) at our institution between December 2017 and June 2018 enrolled in this study. Two patients initially agreed to participate in the study, but due to the discomfort from lying prone for an extended period of time during the biopsy procedure, rescinded their consent during the procedure. There were no exclusion criteria.

### Study Design

**Figure 1:**
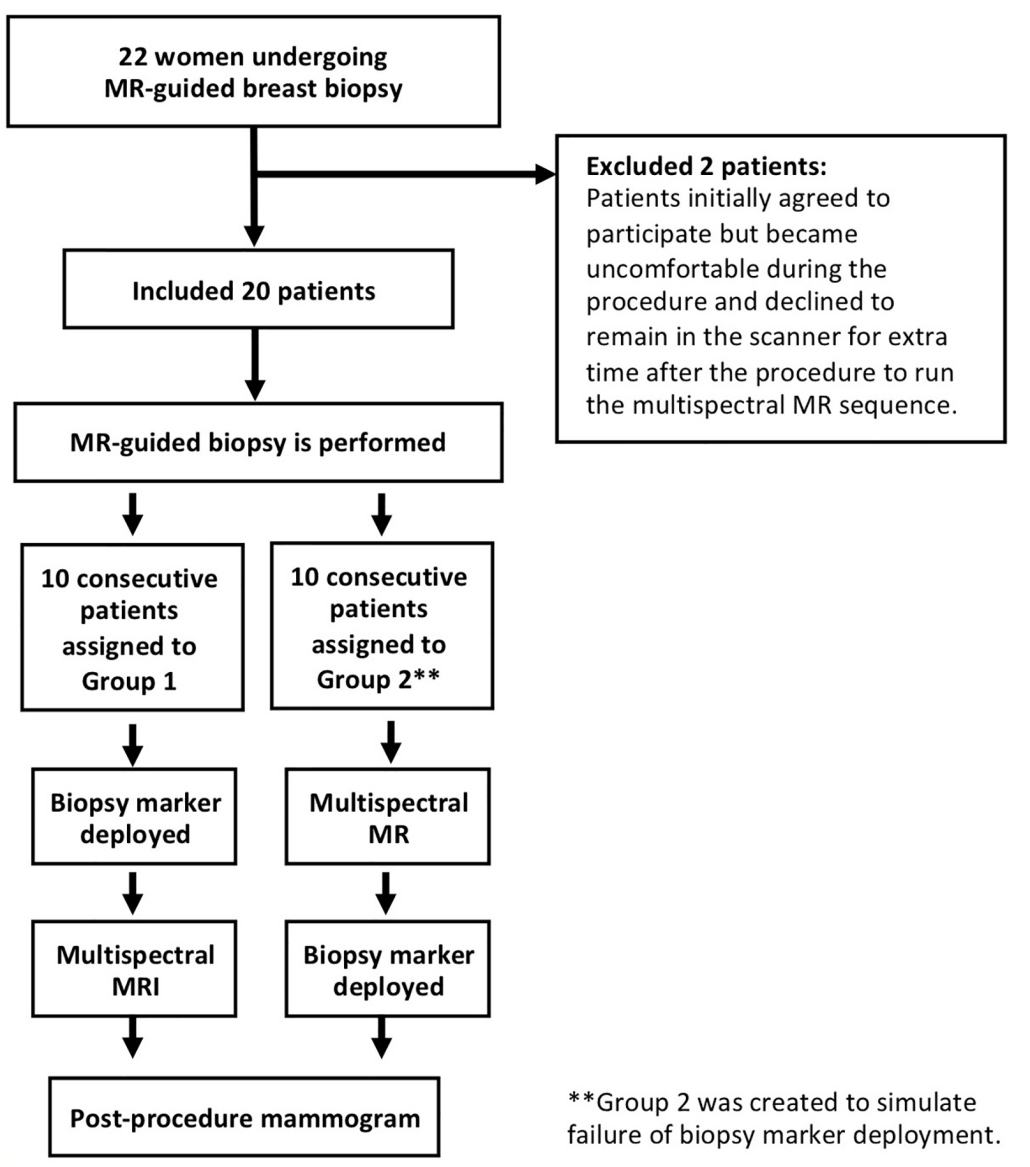
Biopsy marker study design.

Following MRI-guided tissue sampling, a biopsy marker was deployed and a reduced-bin MSI sequence was acquired, although the order of these steps varied. For the first 10 consecutive patients (Group 1), biopsy markers were deployed before MSI. For the last 10 consecutive patients (Group 2), biopsy marker deployment failure was simulated by performing MSI prior to marker deployment (see **Figure 1**). One patient in Group 2 agreed to MSI acquisitions both before and after biopsy marker deployment (although only this patient’s pre-marker MSI images were used in subsequent statistical analysis to maintain consistency). Titanium rod- and hourglass-shaped biopsy markers were used in all patients (Trimark, Hologic, Marlborough, MA). All patients received a same-day post-procedure mammogram.

### Magnetic Resonance Imaging Protocol

All patients were imaged using a 1.5-T scanner (GE Medical Systems, Milwaukee, WI) with an 8-channel breast biopsy coil (Sentinelle, Invivo, Gainesville, FL). A reduced-bin 3D-MSI spin echo technique, adapted from MAVRIC, was employed with Gaussian RF pulses applied at two empirically-selected frequency offsets: +0.7 kHz and +1.5 kHz. No view-angle-tilting was used. A single sagittal slab was prescribed, centered at the expected location of the biopsy marker (TR/TE = 850/17 ms; slice thickness = 2 mm; slice number/encoded sections = 8; FOV = 21 cm; matrix size = 256 × 256; bandwidth = 125 kHz; ETL = 16; sagittal acquisition; Gaussian RF excitation/refocusing pulse bandwidth = 5435/2250 Hz). A 3.1 mT/m selection gradient was applied during excitation to limit the spatial extent of the off-resonance excitation. Scan time for 2-spectral bin (+0.7 kHz and +1.5 kHz) and 1-spectral bin (+0.7 kHz) acquisitions were 2.6 and 1.3 min, respectively.

### Imaging Analysis for Reader Study

Two fellowship-trained breast radiologists (M.R. and K.S., with 10 and 7 years of experience, respectively), blinded both to the mammogram and to the Group 1/Group 2 designations, independently reviewed all patient cases using the MRVIEW software (Mayo Clinic and Foundation). Cases were placed in random order for the review.

**Figure 2:**
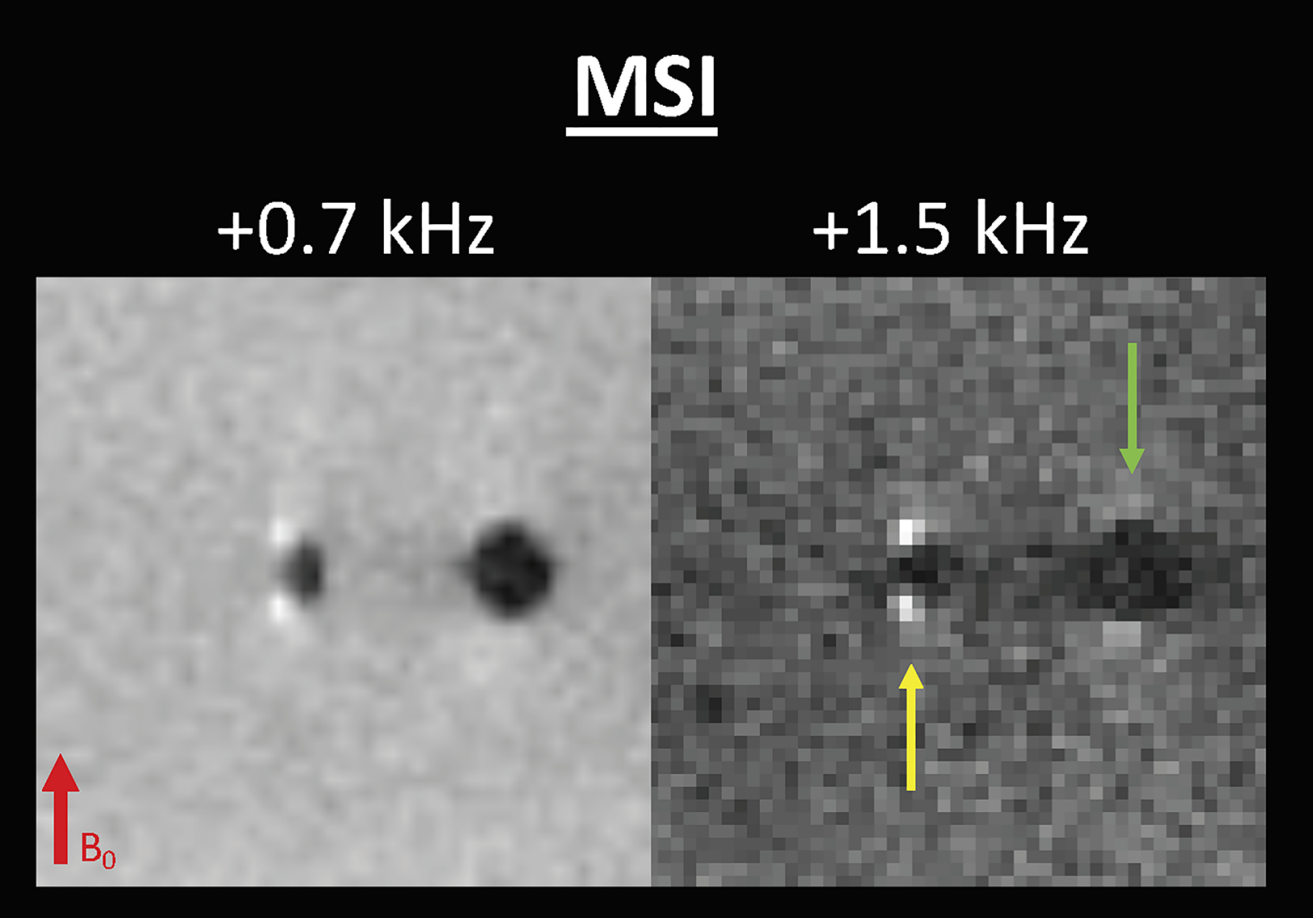
MSI of agarose-based phantom containing a biopsy marker (yellow arrow) and adjacent air (green arrow).

Readers were instructed to identify the location of the biopsy marker by the two bright signal spots adjacent to the marker along the B0 direction, which increased in intensity on the higher frequency offset images. Readers were shown MSI of an agar-based phantom containing air and biopsy markers to teach them the signature biopsy marker pattern (see **Figure 2**).

For each case, readers 1 and 2 first reviewed 1-spectral bin images (+0.7 kHz; scan time 1.3 min) and then the 2-spectral-bin images (+0.7 kHz and +1.5 kHz; scan time: 2.6 min). They evaluated whether the marker was present and, if so, identified its location. Readers identified the presence and location of the markers using the MSI images alone; mammograms were revealed only after these determinations were made. A third reader (S,E, 4 years of experience) then reviewed the MSI images that were marked by readers 1 and 2 as having a biopsy marker present and evaluated whether the biopsy marker location identified on MSI was concordant or discordant with the marker location on mammogram using a 3-point scale: (1) excellent concordance, (2) moderate concordance, and (3) poor concordance.

### Statistical Analysis

The reference standard of this study was the Group 1 and Group 2 designations (which denoted the presence and absence of biopsy markers at the time of MSI) in combination with the mammogram (which confirmed successful biopsy marker deployment for all patients in Group 1).

The sensitivity, specificity, accuracy, positive predictive value (PPV), and negative predictive value (NPV) of 2-spectral-bin and 1-spectral-bin MRI for identification of breast biopsy markers were calculated on a per-patient basis. Exact Clopper–Pearson 95% confidence intervals were computed for all diagnostic accuracy measures to assess the precision of the obtained estimates.

With a Group 1 sample size of 10 patients and a Group 2 sample size of 10 patients, exact Clopper–Pearson 95% confidence intervals for the sensitivity and specificity proportions of interest (i.e., for 2-spectral-bin and 1-spectral-bin; and for each reader, separately) could be constructed to be within ±22.1% of the observed sensitivity and specificity proportions. This calculation assumed anticipated sensitivity and specificity proportions of ≥90%.

The Cohen κ statistic was used to assess inter-rater agreement for the detection of the biopsy marker with MSI (i.e., κ = 0, poor agreement; κ = 0.01–0.20, slight agreement; κ = 0.21–0.40, fair agreement; κ = 0.41–0.60, moderate agreement; κ = 0.61–0.80, good agreement; and κ = 0.81-1.00, excellent agreement). All analyses were performed in SPSS Version 24.0 (IBM SPSS Statistics for Windows, Version 24.0, Armonk, NY: IBM Corp.).

## Results

**Figure 3:**
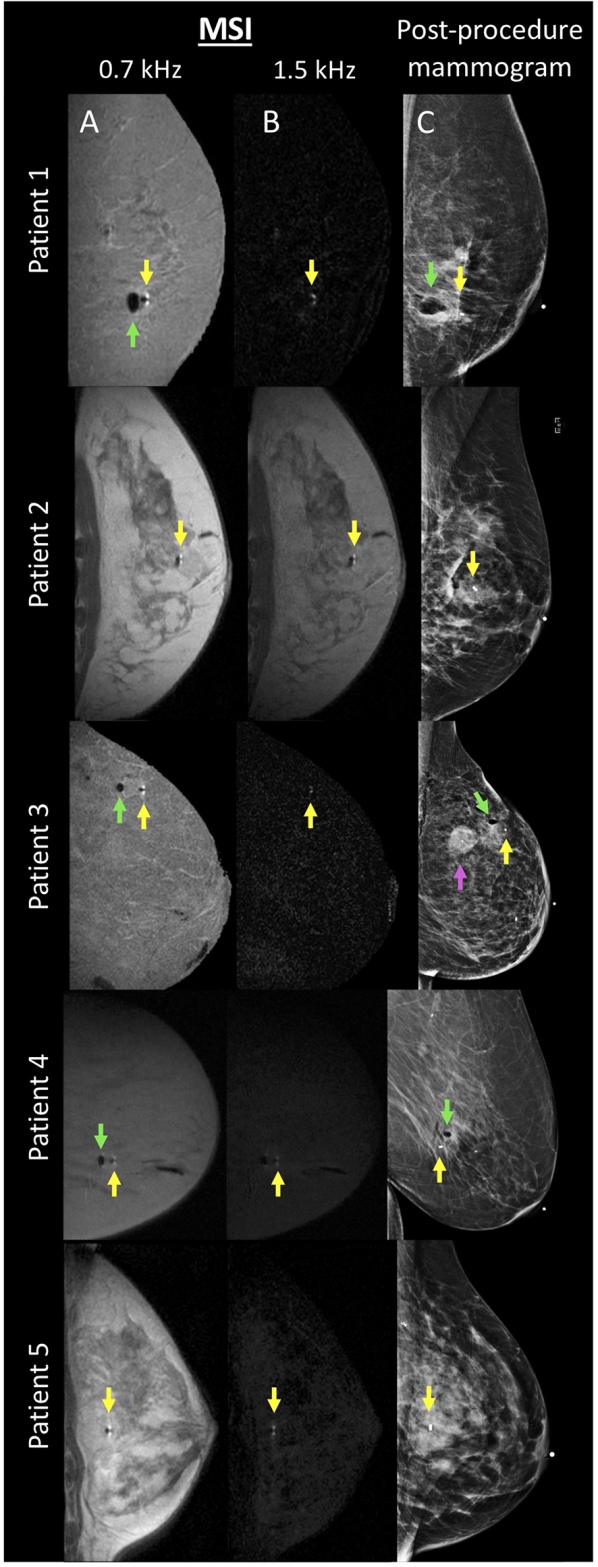
MSI and mammogram performed after MR-guided breast biopsy in 5 patients. For each patient, columns **(A, B)** display the MSI acquired at 0.7 and 1.5 kHz, respectively. Column **(C)** displays the corresponding post-procedure mammogram. Biopsy markers are denoted with a yellow arrow. Adjacent air, if present, is denoted with a green arrow. Patient 3 had a known adjacent tumor, denoted with a magenta arrow.

Twenty female patients undergoing MRI-guided breast biopsy were included in this study (mean age: 57 years; age range: 39–75 years). MSI and post-procedure mammograms were successfully performed and reconstructed for all patients (see **Figure 3**). All post-procedure mammograms documented the location of the biopsy marker.

Using the Group 1 and Group 2 designations in combination with the mammogram as the reference standard, the respective sensitivity, specificity, and accuracy of 2-spectral-bin MSI for readers 1 and 2 for biopsy marker detection were 90.0% (9/10) and 90.0% (9/10), 100.0% (10/10) and 100.0% (10/10), 95.0% (19/20) and 95.0% (19/20) and for 1-bin were 70.0% (7/10) and 80.0% (8/10), 100.0% (10/10) and 100.0% (10/10), 85.0% (17/20) and 90.0% (18/20). Details including PPV and NPV for 1 and 2-spectral-bin MSI are summarized in **Table 1**. Reducing the number of spectral bins from two to one reduced scan time from 2.6 to 1.3 min but also slightly reduced sensitivity. Of note, specificity and PPV remained at 100% for both readers for 1-spectral bin (see **Table 1**).

**Table 1:** Diagnostic accuracy of MSI.

Parameter	TP	TN	FP	FN	Sensitivity	Specificity	PPV	NPV	Accuracy
2-bin MSI									
R1	9	10	0	1	0.90 (0/10) [55.5–99.8]	1.00 (10/10) [69.2–100.0]	100.0 (9/9) [66.4–100.0]	90.9 (10/11) [60.9–98.5]	95.0 (19/20) [75.1–99.9]
R2	9	10	0	1	0.90 (0/10) [55.5–99.8	1.00 (10/10) [69.2–100.0]	100.0 (9/9) [66.4–100.0]	90.9 (10/11) [60.9–98.5]	95.0 (19/20) [75.1–99.9]
1-bin MSI									
R1	7	10	0	3	0.70 (7/10) [34.8–93.3]	1.00 (10/10) [69.2–100.0]	100.0 (7/7) [59.0–100.0]	76.9 (10/13) [56.4–89.6]	85.0 (17/20) [62.1–96.8]
R2	8	10	0	2	0.80 (8/10) [44.4–97.5]	1.00 (10/10) [69.2–100.0]	100.0 (8/8) [63.1–100.0]	83.3 (10/12) [59.1–94.5]	90.0 (18/20) [68.3–98.8]

TP, true positive; TN, true negative; FP, false positive; FN, false negative; PPV, positive predictive value; NPV, negative predictive value.

**Figure 4:**
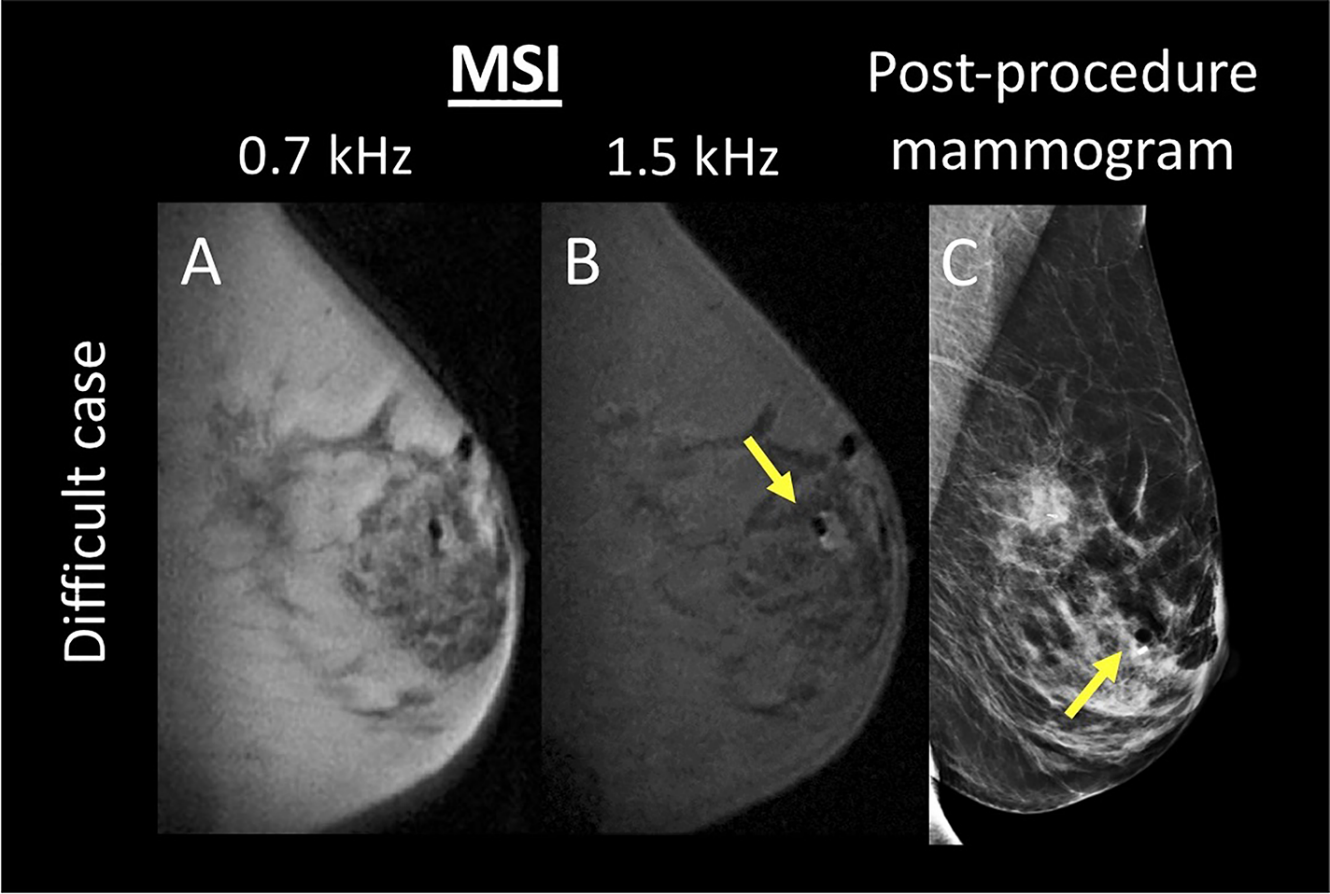
A difficult case where the biopsy marker is within an air pocket, thus partially masking the signature high signal foci that denote biopsy marker location. Both readers failed to identify the presence of the biopsy marker. **(A, B)** display the multispectral images acquired at 0.7 and 1.5 kHz, respectively. **(C)** displays the corresponding post-procedure mammogram. Biopsy marker and air are denoted with a yellow arrow.

There were no false positive cases for either reader for either number of spectral bins. False negative cases occurred when the biopsy marker was within or abutting biopsy cavity air (see **Figure 4**).

For the 9 cases in which readers 1 and 2 identified a biopsy marker, reader 3 evaluated MSI–mammogram concordance on a 3-point scale (excellent, moderate, poor concordance), and found excellent concordance in 100% of cases.

Excellent inter-rater agreement was found using 2-spectral bins (κ = 1.0) and 1-spectral bin (κ = 0.81).

## Discussion

This pilot study demonstrates the feasibility and diagnostic accuracy of multispectral MRI for metallic biopsy marker detection during MRI-guided breast biopsy. MSI may eliminate the need for a post-procedure mammogram, thereby reducing patient discomfort, inconvenience, and ionizing radiation.

In prior work, MRI techniques have been used to visualize paramagnetic markers by compensating for the dipole field with a slice gradient (13), using magnetic signature selective excitation (14), inversion recovery with ON-resonant water suppression (15), or dual-echo projections (16). In this paper, we used an MSI approach. MSI works because metallic biopsy markers exhibit magnetic susceptibilities much greater than that of both air and breast tissue, leading to much more pronounced dipole-pattern magnetic field perturbations. Reduced bin MSI selectively excites the positive lobes of the metallic marker-induced dipole field, creating an image where the biopsy marker is denoted by two bright spots of signal adjacent to the biopsy marker along the B0 direction, i.e., areas of increased magnetic field. MSI has been used in musculoskeletal imaging to map magnetic fields induced by metal hardware (17, 18) (see **Figure 2**). Whereas Quist et al. used an MSI method that enabled improved visualization of underlying anatomy near metallic implants in volunteers with metallic knee, hip, and spinal implants, Shi et al. used MSI to improve the delineation of metallic implant geometry in patients with hip and shoulder replacements. To date, the potential of MSI has not been explored in breast imaging.

A short biopsy marker-detecting MRI protocol would avoid the discomfort of a mammogram as well as shorten the patient’s total procedure time, thus improving the clinical workflow and eliminating the use of ionizing radiation. Minimizing radiation dose is of particular concern in those who undergo repeated MRI breast biopsies, since such patients are often at high breast cancer risk and as such are more sensitive to diagnostic radiation exposure than healthy controls (19, 20).

This feasibility study shows that radiologists are highly accurate in detecting biopsy markers using 2-min MSI. Reducing scan time from 2 min (2-spectral bins) to 1 min (1 spectral bin) maintained specificity and PPV at 100% for both of our readers, while slightly decreasing sensitivity. Both 2-spectral bin and 1-spectral bin protocols are short enough to be easily implemented into a routine MRI-guided breast biopsy protocol without disrupting clinical workflow.

Challenging cases occur when the biopsy marker is within or abuts biopsy cavity air. In such a configuration, air may mask the signature pair of bright foci denoting biopsy marker location and it was in these cases that false negatives occurred. Such cases would benefit from a quantitative modeling approach, where the biopsy marker’s known geometry and magnetic susceptibility are incorporated into a biophysical model to solve for whether or not the biopsy marker is within or abutting an air cavity. This is indeed, the more optimal approach and is a topic of ongoing work. While initial results are promising, it is not yet ready for clinical implementation (21). For now, it will be necessary to acquire a post-biopsy mammogram in these challenges cases.

This work has several limitations. First, it included only a small number of patients; this pilot study warrants further validation with a larger sample size for clinical translation. Second, only two empirically-selected spectral bins were acquired, which may have hampered the diagnostic accuracy of the technique; in future work we will perform experiments to formally optimize the acquired bins and explore post-processing options to optimize performance. This was done to minimize scan time and future work should explore the use of fast 2D MSI techniques to increase the number of bins without increasing the length of exam (11). As our pulse sequence was an adaptation of MAVRIC, we did not use a VAT gradient, although this, as well as the generation of frequency maps and the incorporation of machine learning approaches, will be explored in future work. Finally, MSI was acquired while the breast was still in compression; releasing compression may result in migration of the biopsy marker due to the “accordion effect” (22–24). In the future, MSI should be performed directly after the breast is released from compression, which would better capture the biopsy marker’s final location and create a clinically-feasible alternative to the mammogram.

In this study, we limited our evaluation to the ubiquitous titanium marker, although metallic biopsy markers with a range of material compositions and geometries are also in use. MSI is a suitable technique for detection of any metallic biopsy marker and would require only minor adjustments in RF pulse frequency offsets that are proportional to the magnetic susceptibility of the marker material to be imaged.

In conclusion, MSI is a feasible, diagnostically accurate technique for identifying metallic biopsy markers during MRI-guided breast biopsy and may eliminate the need for a post-procedure mammogram.

## Data Availability Statement

The datasets presented in this article are not readily available because all of the data is not currently in a completely deidentified format. Data will be shared with qualified researchers whose proposed use of the data has been approved. Requests to access the datasets should be directed to SE-W (eskreiss@mskcc.org).

## Ethics Statement

The studies involving human participants were reviewed and approved by the Institutional Review Board, Weill Cornell Medicine. The patients/participants provided their written informed consent to participate in this study.

## Author Contributions

All authors contributed to the conception or design of the work, the acquisition, analysis, or interpretation of data for the work, and the drafting of the work or revising it critically for important intellectual content. All authors contributed to the article and approved the submitted version.

## Funding

This research was funded in part through the National Institutes of Health (S10 OD021782; Clinical and Translational Science Center at Weill Cornell Medical College 1-UL1-TR002384-01; and NIH/NCI Cancer Center Support Grant P30 CA008748) and Susan G. Komen.

## Conflict of Interest

YW and PS are inventors on QSM-related patents issued to Cornell University and hold equity in Medimagemetric LLC. KP received payment for activities not related to the present article including lectures including service on speakers bureaus and for travel/accommodations/meeting expenses unrelated to activities listed from the European Society of Breast Imaging (MRI educational course, annual scientific meeting), and the IDKD 2019 (educational course). EM has received a grant from GRAIL, Inc. for research not related to the present article.

The remaining authors declare that the research was conducted in the absence of any commercial or financial relationships that could be construed as a potential conflict of interest.
